# Cardiorespiratory fitness levels and associations with physical activity and body composition in young South African adults from Soweto

**DOI:** 10.1186/s12889-017-4212-0

**Published:** 2017-04-05

**Authors:** A. Prioreschi, S. Brage, K. Westgate, S. A. Norris, L. K. Micklesfield

**Affiliations:** 1 MRC/WITS Developmental Pathways for Health Research Unit, Department of Paediatrics, School of Clinical Medicine, Faculty of Health Sciences, University of Witwatersrand, Johannesburg, South Africa; 2 MRC Epidemiology Unit, University of Cambridge, Cambridge, UK

**Keywords:** Fitness, Physical activity, Sedentary behaviour, Accelerometry, Soweto

## Abstract

**Background:**

This observational study aims to describe fitness, and objectively measured physical activity levels and patterns in 409 young black South African adults (aged 19–20 years) from Soweto, as well as to examine associations between physical activity, fitness and BMI.

**Methods:**

A sub-maximal ramped step test was used to obtain an estimate of maximal oxygen uptake (VO_2max_). Physical activity was measured using ActiGraph (GT1M) for 7 days in 256 participants. Time spent in sedentary (<100 counts per minute (cpm)), moderate (2020–5998 cpm) and vigorous (≥5999 cpm) intensity activity was calculated, and 90% of participants were considered active. Data are presented as mean(CI) or median(CI).

**Results:**

Overweight and obesity was more prevalent in females than males (35% vs 8%, *p* < 0.001). Males had a higher VO_2max_ than females (41.9(41, 43) vs 32.6(32, 33)mlO_2_/kg/min, *p* < 0.001); spent more time in moderate to vigorous intensity physical activity (MVPA) (83(80, 94) vs 43(38, 45)min/day, *p* < 0.001), and less time in sedentary behaviours (541(541, 567) vs 575(568, 597)min/day, *p* < 0.01). Sedentary time was not associated with VO_2max_, however BMI was inversely associated, and MVPA was positively associated, with VO_2max_ (both *p* < 0.001).

**Conclusions:**

The majority of young South African adults in this study were sufficiently active, and higher MVPA was associated with fitness. However, the high level of sedentary behaviour in this population is of concern and may be contributing to the increasing prevalence of overweight and obesity in this population. Young South African females are at greatest risk for decreased cardiovascular fitness and should be the focus for future interventions.

**Electronic supplementary material:**

The online version of this article (doi:10.1186/s12889-017-4212-0) contains supplementary material, which is available to authorized users.

## Background

South Africa is undergoing rapid urbanisation and experiencing consequences typical of a transitioning society, such as increased physical inactivity and obesity [1]. Self-report physical activity data from the 2010 World Health Organisation (WHO) global health status report [2] have shown that 55.7% of female and 46.4% of male adolescents were inactive according to American College of Sports Medicine (ACSM) guidelines (150 min of moderate activity or 60 min of vigorous activity per week). Furthermore, 58.5% of males and 71.8% of females over the age of 20 years were overweight [2]. Soweto is an urban township in South Africa that clearly exhibits these transitions, with women showing particularly high and increasing rates of overweight and obesity. The increasing prevalence of overweight and obesity, as well as physical inactivity in South Africa [3], makes it extremely important to start examining correlates of obesity and physical activity in young adults, which is a time when these trajectories are being set up [4].

Increased cardiorespiratory fitness, has been associated with decreased overall mortality and morbidity, independent of other risk factors such as high body mass index (BMI) [5]. Fitness data in South Africa is limited, particularly in young adults. The South African National Health and Nutrition Examination Survey (SANHANES) data in 2013 show that only 66% of males and 38% of females (18–24 year old) were considered fit [1]. Fitness and physical activity have been associated with improved cardiovascular and metabolic health; as well as reduced adiposity in adults, adolescents and children [6, 7].

The associations between fitness, habitual physical activity levels and BMI in young South African adults is not well understood. Furthermore, the factors that influence fitness in this population have not yet been fully described. Therefore, this study aims to describe fitness and physical activity levels and patterns in a group of young black South African adults from Soweto, and to examine the associations between fitness, habitual physical activity and BMI.

## Methods

### Participants

Participants for this cross sectional study were recruited through the Birth to Twenty (BT20) cohort study conducted in Soweto, Johannesburg, which has been described in detail previously [8]. The original sample followed up 3273 children born in 1990 (April to June) from birth. For this study, 423 randomly selected adolescents were included between December 2009 and October 2010 (aged 19–20). Of the 423 participants who were enrolled in the study, 409 provided a valid fitness estimate, and 306 wore an accelerometer. After applying wear time inclusion criteria of ≥500 min of wear time per day and ≥3 valid days, 256 participants (119 females and 137 males) had valid physical activity data as well as a valid fitness estimate. Ethical approval was obtained from the Human Research Ethics Committee of the University of the Witwatersrand (ethics number M091016). All participants gave written and informed consent for participation in this study.

### Measurements

Height was measured in meters using a standard stadiometer (Holtain). Weight was measured to the nearest 0.1 kg using a standard digital scale (Dismed, USA) by trained research staff with participants barefoot and wearing minimal clothing, and BMI was calculated as weight(kg)/height(m)^2^. BMI categories for sex were defined according to WHO criteria [2] and expressed as underweight (<18.5 kg/m^2^), normal weight (≥18.5 and <25 kg/m^2^), overweight (≥25 kg/m^2^), or obese (≥30 kg/m^2^).

Cardiovascular fitness was estimated using a ramped submaximal step test, which has been described elsewhere [9]. Briefly, following a short medical screening questionnaire, participants were asked to step up and down on a standard exercise step (Reebok, 20 cm) for eight minutes to a voice prompt, followed by a two minute seated recovery. Prior to the test, participants were asked to sit quietly for 10 min, following which their resting heart rate was recorded using a heart rate monitor. During the step test and recovery period, electrocardiogram (ECG) and acceleration waveform data was recorded continuously using a combined heart rate and movement sensor (ActiHeart, CamNtech, Papworth UK). The step test was discontinued if the participant was unable to keep the correct pace, started to lose balance, or verbally stated that they wanted to discontinue.

ECG data was visually reviewed and noisy data was masked from analysis. Heart rate data was summarised in 15-s intervals. All tests ≥4 min were eligible for inclusion. Heart rate (above resting values) during the step test was regressed against predicted workload and combined with recovery heart rate, resting heart rate and test duration parameters to determine the individual’s submaximal response, which was extrapolated to age-predicted maximum heart rate [10] in order to obtain an estimate of maximal oxygen uptake (VO_2max_) in mlO_2_/kg/min [11]. Fitness levels were determined using sex specific medians, where participants above or equal to the 50th percentile for their sex were classified as ‘fit’ and those below the 50th percentile were classified as ‘unfit’.

Objective physical activity was measured using an accelerometer (ActiGraph GT1M, Pensacola, FL) worn on the right hip for 7 days, initialised to record data at 5-s intervals. Participants were asked to wear the accelerometer at all times during the day for 7 days (except when bathing or sleeping), whilst continuing with their usual behaviour. Following the measurement, the monitor was returned and downloaded. Non-wear time was defined as >90 min of continuous zero counts. Following removal of non-wear time, a minimum of 500 min of wear time was required for a day to be considered valid. At least 3 valid days were required for inclusion in the analysis. Since some participants wore the accelerometer while sleeping, any data recorded between 12 pm and 6 am was considered sleep time and was removed. Total physical activity (counts per minute (cpm)) as well as time spent (minutes per day) in different intensity categories were calculated: sedentary activity was defined as time spent <100 cpm, light intensity activity as time between 100 and 2019 cpm, moderate intensity activity as time between 2020 and 5998 cpm, and vigorous intensity activity as time ≥ 5999 cpm [12]. MVPA was calculated as the sum of minutes in moderate and vigorous activity per day. Participants were classified as ‘active’ if their average daily activity levels equated to at least one seventh of the recommended weekly levels (150 min of moderate to vigorous intensity activity per week and/or 75 min of vigorous intensity activity per week) according to the Centre for Disease Control (CDC) recommendations [13]. Participants who did not meet these criteria were classified as ‘inactive’. For comparison with the WHO physical activity recommendations, 10-min activity bouts were defined as 10 or more consecutive minutes above the relevant threshold [14]. Participants were considered to have met these recommendations if they accumulated an average of 30 min of moderate or higher intensity activity accumulated in 10-min bouts (3 bouts) per day.

All statistical analyses were completed using Stata13 for Mac. All continuous data are presented as mean(SD), or median(CI) for non-parametric data. Data were stratified by sex and descriptive data were compared between males and females using Student’s t tests for parametric data and Mann-Whitney U tests for non-parametric data. Ordinal data were compared using Chi-squared tests. Univariate linear regressions were completed to determine associations between VO_2max_ and the exposure variables. Thereafter, a multiple regression was completed to determine predictors of VO_2max_ using significant linear correlates (BMI, height and weight, MVPA, sedentary time, and sex), and excluding any collinear variables (weight and height). Regression margins were calculated by regressing BMI and fitness category on MVPA. ANOVA was used to determine differences in estimated VO_2max_ according to BMI category for males and females separately, with a Bonferroni post hoc test. Significance was set at *p* < 0.05.

## Results

Participant characteristics are presented in Table 1, stratified by sex. Data for all participants with fitness results (*n* = 409) are presented in all cases except for the physical activity variables where data for only 256 participants with valid accelerometer data are shown. There were no differences between participants with valid physical activity data compared to those without (Supplementary Table 1). Males were significantly taller and had a lower BMI than females. The majority (75%) of the males were classified as having a normal BMI, while 26% of the females were classified as overweight and 9% as obese, compared to only 6% and 2% of the males respectively (*p* < 0.001).

**Table 1:** Participant characteristics by sex

	Male (*n* = 218)	Female (*n* = 191)	*P* value
Weight (kg)	62.5 (61.1, 63.8)	61.3 (59.4, 63.2)	0.32
Height (m)	1.72 (1.71, 1.73)	1.60 (1.59, 1.61)	<0.001*
BMI (kg/m^2^)	21.02 (20.6, 21.4)	23.85 (23.2, 24.6)	<0.001*
BMI category (%)			<0.001*
Underweight	17	12	
Normal	75	53	
Overweight	6	26	
Obese	2	9	
Fitness			
VO_2max_ (mlO_2_/kg/min)	41.9 (41.3, 42.6)	32.6 (31.9, 33.2)	<0.001*
Physical Activity	Male (*n* = 137)	Female (*n* = 119)	
Physical activity (cpm)	549 (525, 603)	306 (290, 327)	<0.001*
Sedentary (min/day)	541 (541, 567)	575 (568, 597)	<0.01*
Light intensity (min/day)	128 (126, 141)	132 (126, 140)	0.69
Moderate intensity (min/day)	78 (73, 85)	42 (37, 43)	<0.001*
Vigorous intensity (min/day)	5 (6, 9)	1 (1, 1)	<0.001*
MVPA (min/day)	83 (80, 94)	43 (38, 44)	<0.001*
Active CDC (%)			<0.001*
Active	96	83	
Inactive	4	17	
Active WHO (%)			<0.001*
Active	40	20	
Inactive	60	80	

*p < 0.01 between groups

Data are mean(CI), or median(CI) for physical activity variables unless otherwise stated

*BMI* Body mass index, *cpm* counts per minute, *MVPA* Moderate to vigorous physical activity

**Fig. 1:**
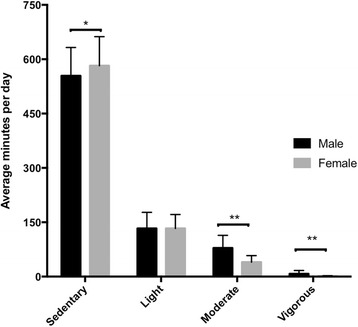
Distribution of daily physical activity between males and females. *p < 0.01. **p < 0.001

Males had a higher estimated VO_2max_, and significantly more males were classified as active (96% of males vs 83% of females using the CDC guidelines, *p* < 0.001) compared to females. Time spent in both moderate and vigorous intensity physical activity was significantly higher (both *p* < 0.001), while sedentary time was significantly lower (*p* < 0.01), in males than females (Fig. 1). The majority of the participants (90%) met the CDC recommendations for physical activity, however when WHO guidelines, which include activity bouts, were assessed the percentage of participants meeting guidelines decreased to 40% in males and 20% in females. Significantly more males than females were classified as active using both CDC and WHO criteria (both *p* < 0.001).

Results of the multiple regression are presented in Table 2. Higher MVPA, lower BMI, and sex (being male) were all significantly associated with higher VO_2max_, however sedentary time was not associated with fitness (R^2^ for the model =0.60). This analysis was powered at a level of 1.0. Regression means for time in MVPA were 73 min/day for fit individuals, and 58 min/day for unfit individuals, and regression means for BMI were 21.35 kg/m^2^ for fit and 23.41 kg/m^2^ for unfit individuals.

**Table 2:** Multiple linear regression results for significant linear exposures regressed on the fitness outcome (VO_2max_)

	Coefficient	*p* value	95% CI
Sedentary (min/day)	0.00	0.42	−0.00, 0.01
MVPA (min/day)	0.03	<0.001*	0.02, 0.05
BMI (kg/m^2^)	−0.30	<0.001*	−0.42, −0.17
Sex (male)	7.52	<0.001*	6.23, 8.82

R^2^ for this regression = 0.60

*p < 0.001

BMI – Body mass index, MVPA – moderate to vigorous physical activity

**Fig. 2:**
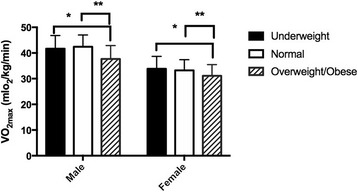
Distribution of estimated VO_2max_ by BMI category for males and females

Estimated VO_2max_ according to BMI category is presented in Fig. 2, for males and females separately. In both males and females, estimated VO_2max_ was higher in underweight compared to overweight and obese participants (*p* < 0.05), and higher in normal weight compared to overweight and obese participants (*p* < 0.0001).

## Discussion

This study aimed to describe fitness and physical activity levels in young black South African adults from an urban township in Johannesburg (Soweto), as well as to determine whether fitness is associated with physical activity levels and BMI in this population. Significant and independent associations were found between MVPA and fitness, as well as between BMI and fitness. The majority of participants (90%) met the physical activity guidelines without prescriptions for bouted activity, yet only 40% met the guidelines for bouted activity. Both fitness, and time spent in moderate and vigorous intensity physical activity, were significantly higher in males than females. Females spent significantly more time in sedentary behaviours, although sedentary time was high (mean of 9 h and 20 min) for all participants. BMI was also significantly higher in females than males, and so it can be concluded that females in this population are at greatest risk of disease consequences associated with reduced fitness, high levels of physical inactivity and a high prevalence of overweight and obesity.

The present study, in concurrence with many others [12, 15, 16], reported that males are more physically active than females. In both males and females, time spent in MVPA was positively associated with fitness, where fit individuals spent 15 more minutes per day in MVPA than unfit individuals, yet sedentary time was not associated with fitness. SANHANES data showed that 66% of 18–24 year old males were fit, while only 38% of females of the same age were classified as fit, as assessed using a three minute submaximal step test [1]. These percentages varied by location and race, and physical activity levels were not reported. Objectively measured energy expenditure data from the Physical Activity and Health Longitudinal (PAHL) study in South Africa reported that 16 year old girls were more active than boys, spending 61 min/day in MVPA compared to 35.0 min/day in boys. However, the results may have been influenced by the higher fat mass present in girls and fitness was not assessed in the PAHL participants [17]. There is a paucity of data examining associations between physical activity and fitness in young adults, specifically in South Africa, and differences in measurements used makes comparison of the available data difficult. Data from the Helena study in younger European adolescents (aged 12–18 years) [18], reported similar findings to ours as higher fitness levels (as assessed using a 20 m shuttle test), were associated with higher physical activity levels and less time spent sedentary. Adults (aged 18–49 years) from the National Health and Nutrition Examination Survey (NHANES) database had similar fitness levels to our participants assessed using a submaximal treadmill test (42.6 vs 41.9 mlO_2_/kg/min for males and 35.7 vs 32.6 mlO_2_/kg/min for females). In the NHANES data, fitness was associated with MVPA, but not sedentary time [19].

The observed differences in fitness between males and females in the present study, besides being associated with physical activity levels, may also be influenced by differences in BMI. Similarly to what has been shown in other studies, BMI was inversely associated with estimated VO_2max_. We have also shown significant differences in BMI between males and females and reported a high prevalence of overweight and obese females; however this prevalence is still lower (8% compared to 10% in males, and 35% compared to 47% in females) than that reported in the SANHANES data on young South African adults (18–24 years) [1]. Approximately 15% of participants in this study were underweight, yet VO_2max_ was significantly higher in underweight compared to obese males and females. This concurs with current literature which shows that low and normal BMIs have beneficial effects on fitness in adolescents and young adults [20, 21], likely due to the bidirectional associations between functional respiratory impairment and increased energy expenditure requirements from overweight and obesity [22, 23], but also due to the size dependant nature of the measure.

In this cohort of urban black young adults 96% of males and 83% of females met the recommended CDC physical activity guidelines of 150 min MVPA per week or 75 min vigorous activity per week. However, when using the WHO recommendations that require this activity to be performed in bouts of a minimum of 10 min, only 40% of males and 20% of females were classified as physically active. From these data it would suggest that these young adults are accumulating sufficient amounts of MVPA throughout the day, yet are accumulating this activity sporadically rather than in bouts of 10 min or more. Recent studies using NHANES data have shown that when comparing bouts of moderate and vigorous activity to total accumulated moderate and vigorous activity, bouted activity infers greater benefits on adiposity, yet has similar benefits as accumulated activity on cardiometabolic risk factors [24, 25]. Conversely to our findings, self-report data from previous South African studies presented in the WHO global health status report have shown that 46% of male adolescents and 56% of female adolescents 1–2 years younger than our cohort are inactive when using similar guidelines to the CDC recommendations [2]. Self-report SADHS data from 19 year old urban South Africans have also shown that 49% of males and 75% of females were inactive [3] using GPAQ (WHO) classifications. It is possible that the peri-urban setting from which the present cohort was drawn resulted in higher levels of physical activity for commuting purposes contributing to the accumulation of the recommended amount of MVPA per week, yet lower levels of structured leisure time physical activity thus resulting in sporadic accumulation of higher intensity activity throughout the day. Indeed, the SADHS data showed lower inactivity levels in non-urban vs urban females (75% vs 82% inactivity), but reported no differences for males. It is also possible that discrepancies between self-report and objectively assessed data could be affecting these comparisons. Self-report physical activity data is prone to inaccuracy, and the objective assessment of physical activity levels and patterns in the present study should be considered a strength.

Although the majority of this sample met unbouted physical activity recommendations, sedentary time equated to an average of just less than 9 and a half hours per day (>50% of waking hours) for both groups, while time spent in vigorous intensity activity equated to less than 1% of the day. To our knowledge, this is the only data reporting objectively measured sedentary time in this age group in Soweto. One study in older adult females (mean age 41 years) from Soweto showed that sedentary time was lower - on average 3 h per day; and was not different between those women classified as physically active vs inactive [26]. Furthermore 90% of women in this study did not perform any vigorous intensity activity. However, the 2016 Healthy Active Kids South Africa report has shown that children up until the age of 17 are exceeding sedentary recommendations, and that sedentary time (largely assessed as screen time) increases with age [27]. This high sedentary time in our study is similar to what has been found in European adolescents from the HELENA study [18], who spent 9 h per day being sedentary, but higher than findings from American adolescents and young adults who are reported to spend 7–8 h per day sedentary [28]. With regard to physical activity patterns, similar to the current findings, previous self-report data from young (26 ± 7 years) South African women showed no time reported in vigorous intensity activities per week [7]. Vigorous activity is likely to be accumulated during leisure time physical activity or sports participation, rather than during general daily activities. This implies low levels of voluntary high intensity physical activity participation in this population, confirming findings from studies in other South African cohorts [7].

**Fig. 3.**
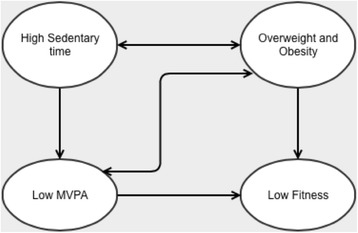
Proposed bidirectional relationship between sedentary time, physical activity, BMI and fitness

This high sedentary time and low amounts of vigorous intensity activity, in combination with a high prevalence of overweight and obesity; and the relationships with fitness levels puts these young adults at risk of future cardiometabolic disease. This relationship is most likely bidirectional, where high levels of sedentary behaviour and low fitness results in excess adiposity, thereby increasing BMI. Increased adiposity likely results in further increases in sedentary time, potentially at the expense of moderate and vigorous intensity activity and lower fitness (illustrated in Fig. 3). This vicious cycle of deleterious effects requires intervention, seemingly directed at increasing MVPA; and decreasing BMI and sedentary time, which may allow for improvements in fitness and thus cardiometabolic health. Particular focus should be placed on targeting young female adults, who are at increased risk due to their lower physical activity and fitness levels, higher sedentary time, and high prevalence of overweight and obesity. Considerations of the population and gender specific barriers and factors that could affect interventions should also be made [29, 30]. The findings of this study highlight the importance of increasing MVPA for improvements in fitness; and previous studies have also shown increases in fitness in relation to increases in physical activity (particularly vigorous intensity activity) in populations of similar ages [31, 32]. However, the importance of decreasing sedentary time should not be overlooked. In conjunction with walking for transport, which is already common in this population but tends to be performed at lower intensities, young South African women should also be encouraged to participate in structured, high intensity physical activity on most days of the week, and to reduce sedentary time as much as possible. Potentially, this physical activity should be accumulated in bouts of 10 min of more in order to confer maximum beneficial effects on body composition, thus allowing for improved fitness.

Limitations of this study include the cross sectional design, which limits any causality conclusions. Furthermore, the lack of detailed body composition data prevents deeper analysis into possible correlates of fitness in this cohort, although BMI is a commonly used proxy for body composition. Although the objective measurement of physical activity is a strength of this study, more detail into the context of these activity behaviours would have also been beneficial. The limited number of participants with complete accelerometer data decreased the sample size for physical activity analysis, yet these analyses were still sufficiently powered.

## Conclusions

In conclusion, the majority of young South African adults in this study are sufficiently active, yet are not accumulating activity in prolonged bouts, which may be detrimental to body composition. Moderate to vigorous intensity physical activity and BMI are both strongly and independently associated with fitness. The large amount of time spent in sedentary behaviour observed in these young adults is of concern and should be a major focus of any intervention; along with increasing structured, higher intensity activity and decreasing BMI, and focus should be placed on young adult females.

## Additional file

Additional file 1: Table S1. Comparison of participants with complete accelerometer data to the larger sample of participants with only fitness data”, which provides data comparing anthropometric and fitness data for the participants with complete acceletometer data to those with only fitness data. (DOCX 52 kb)

## Abbreviations

ACSM: American college of sports medicine
BMI: Body mass index
CDC: Centre for disease control
Cpm: Counts per minute
ECG: Electrocardiogram
GPAQ: Global physical activity questionnaire
MVPA: Moderate to vigorous physical activity
NHANES: National health and nutrition examination survey
PAHL: Physical activity and health longitudinal (study)
SADHS: South African demographic and health survey
SANHANES: South African national health and nutrition examination survey
WHO: World health organisation
