# Supplementary material for: Cardiorespiratory fitness levels and associations with physical activity and body composition in young South African adults from Soweto
Source: BMC Public Health. 2017 Apr 5;17:301. doi: 10.1186/s12889-017-4212-0 (PMC5382390; doi:10.1186/s12889-017-4212-0)
Supplement: Additional file 1: Table S1. — Comparison of participants with complete accelerometer data to the larger sample of participants with only fitness data”, which provides data comparing anthropometric and fitness data for the participants with complete acceletometer data to those with only fitness data. (DOCX 52 kb) [file 12889_2017_4212_MOESM1_ESM.docx]

Supplementary Table 1. Comparison of participants with complete accelerometer data to the larger sample of participants with only fitness data

|  | **Complete activity and fitness data (n=256)** | **Fitness data only (n=153)** | **P value** |
| --- | --- | --- | --- |
| Weight (kg) | 61.7(60.2, 63.2) | 62.2(60.5, 64.0) | 0.66 |
| Height (m) | 1.66(1.66, 1.69) | 1.66(1.65, 1.67) | 0.31 |
| BMI (kg/m^2^) | 22.34(21.79, 22.89) | 22.35(21.68, 23.01) | 0.99 |
| **Fitness** |  |  |  |
| VO_2max_ (mlO_2_/min/kg) | 37.25(36.20, 38.31) | 37.73(36.92, 38.54) | 0.48 |

Data are mean(CI) unless otherwise stated.

BMI – Body mass index
